# Prediction of body composition in mirror carp (*Cyprinus carpio*) by using linear measurements in vivo and computed tomography post-mortem

**DOI:** 10.5194/aab-63-69-2020

**Published:** 2020-02-25

**Authors:** Paula Maas, Beata Grzegrzółka, Philipp Kreß, Martin Oberle, Michael Judas, Prisca Valerie Kremer-Rücker

**Affiliations:** 1 Faculty of Agriculture, University of Applied Sciences Weihenstephan-Triesdorf, Steingruberstraße 2, 91746 Weidenbach, Germany; 2 Faculty of Animal Science, Department of Genetics and Animal Breeding, Warsaw University of Life Sciences – SGGW, Ciszewskiego 8, 02-786 Warsaw, Poland; 3 Bavarian State Institute of Fisheries, Greiendorfer Weg 8, Höchstadt an der Aisch, Germany; 4 Max-Rubner-Institut, Department of Safety and Quality of Meat, E.-C.-Baumann-Straße 20, 95326 Kulmbach, Germany; 5 Faculty of Veterinary Medicine, Ludwig-Maximilians-Universität München, Chair of Food Safety, Schönleutnerstraße 8, 85764 Oberschleissheim, Germany

## Abstract

The mirror carp (*Cyprinus carpio*) belongs to the cyprinids, the world's largest and most important fish family in aquaculture. The fat content and the fillet yield are important parameters in the marketing of carp. Although the influence of the environment on the body composition of the carp has been well studied, there is little research in the field of breeding. For this purpose, precise phenotyping is indispensable. Therefore, during this study a total of 33 mirror carps were examined using computed tomography (CT) technology. First, the fish were examined alive. Total body weight and linear measurements such as lengths, height and circumferences were measured, and ultrasound was used to determine the back-fat thickness. The fish were then slaughtered and whole body scans of all fish using CT were made. The carps were filleted and the fillets with skin were chemically analyzed.

In order to predict the chemical fillet fat content, thickness measurements and volume calculations of the back fat were carried out using CT. Compared to the CT-based back-fat thickness measurement correlated with the results from the chemical analysis ($R^{2}=0.62$), the CT-based volume measurement of the back fat leads to a higher coefficient of determination ($R^{2}=0.85$). Prediction results can still be improved by adding linear measurements. The in vivo ultrasound (US) examination of the back-fat thickness was compared with the CT back-fat thickness results. The measurements of the back-fat thickness took place at similar positions in the fish. Coefficients of determination ($R^{2}$) of 0.63 to 0.77 were obtained. The back fat in mirror carp proved to be an interesting area for determining the fillet fat content.

The evaluation of the fillet yield resulted in a mean value of 42.89 % with a standard deviation of ±2.43. Fillet yield (%) correlated with CT-based fillet thickness measurement resulted in a moderate coefficient of determination ($R^{2}$ of 0.45). A similar coefficient of determination was achieved with selected linear measurements.

## 1 Introduction

Aquaculture will play a major role in feeding the growing world population, which will mean feeding nearly 10 billion people by 2050 – in this context aquaculture is of particular interest when it comes to solving the upcoming global protein deficiency (Evans, 2009; Searchinger et al., 2018). Worldwide, cyprinids are the most important and largest fish family. The common carp (*Cyprinus carpio*) is the third most produced species in aquaculture (FAO, 2018). It is a very sustainable fish, which is typically kept under extensive or semi-intensive conditions. Therefore, the environmental conditions have a huge impact on the performance of carp, and pond management has been a major focus of attention (Horváth et al., 2008). Breeding is a complementary way to produce tasty carps. For the selection of suitable parents a method is required to analyze the carcass quality in vivo.

Different imaging technologies such as ultrasound (US), magnetic resonance imaging or computed tomography are used for performance testing in live animals (Scholz et al., 2015). Computed tomography (CT) has already been successfully used for determining carcass composition in fish species such as salmon, rainbow trout, cod, common carp, grass carp and silver carp (Gjerde, 1987; Rye, 1991; Romvári et al., 2002; Hancz et al., 2003b; Kolstad et al., 2004, 2008). This imaging technology is based on the density-related attenuation of x-rays by different tissues. The object to be examined is positioned on a table and moved stepwise through the gantry of the CT device. A measuring unit consisting of an x-ray tube and opposing detectors rotates along the gantry. During rotation, the object is irradiated and the remaining radiation after passing the object is detected. From these measurements, a specific attenuation value is calculated for each volume element (voxel). The attenuation is expressed in Hounsfield units (HU) and can range from -1000 HU for air to +1000 HU for very dense tissues (Scholz et al., 2015).

Fat content and fillet yield are the main traits in the marketing of carp. Previous studies investigated the morphology (Cibert et al., 1999) and heritability estimates of the fillet yield (heritability of 0.38 for % fillet yield with skin; Kocour et al., 2007). Regarding the fat content, a high variation has been described in common carp: Zeitler et al. (1984) 6.7 %–17.6 %, Ljubojević et al. (2013) 6.3 %–15 %, Bauer and Schlott (2009) 2.7 % to 6.9 %.

Oberle and Aas (2015) described negative effects on the taste of carp flesh exceeding a fat content of 15 %. In Germany, some areas are allowed to produce carp under a European Quality Scheme (EU Regulation no. 1151/2012 article 7, 2012). According to the requirements laid down in the product's specifications, the fat content including the skin should not exceed 10 %. Due to the fact that carps are mostly traded alive, fast and noninvasive methods are needed to determine carcass traits. Currently the fat content is determined by a microwave-based Fish Fatmeter (Kent, 1990). Oberle et al. (2015) reported a coefficient of determination of $R^{2}=0.85$ between the back-fat thickness, measured at the split carcass, and the fat content measured by the Fish Fatmeter. During a previous study, the back-fat thickness of carps was measured by ultrasound and showed moderate coefficients of determination with the fat content measured by the Fish Fatmeter ($R^{2}=0.41$) (Maas et al., 2015).

The measurement of back-fat thickness with an ultrasound is widely used for other species, such as pigs and cattle, as a noninvasive method to determine the carcass quality (Brethour, 1992; Newcom et al., 2002; Müller and Polten, 2004). It is also possible to estimate various parameters in animals using linear measurements. In cattle, linear measurements are successfully used to predict body weight (Alderson, 1999; Ozkaya and Bozkurt, 2009).

The objective of the present study was to estimate the body composition and fillet yield of carps using computed tomography and different linear body measurements. Chemical analysis and dissection were used as reference methods. In addition, a validation of the back-fat thickness measurement by ultrasound was done.

## 2 Material and methods

### 2.1 Animals

During autumn 2014, 33 three-year-old mirror carps (*Cyprinus carpio*) from six different ponds in Bavaria, southern Germany, were examined for their body composition. The carps were reared in fish ponds with semi-intensive polyculture based on natural feed and supplementary feed in the form of cereals. The measurements occurred during the regular harvesting. The netted, live carps were measured for their back-fat thickness using a mobile ultrasound device, followed by weighing and measuring of different linear parameters. The fish were then slaughtered and full-body CT scans were performed. After the CT scan, the fish were filleted and one fillet including skin was analyzed chemically.

The Bavarian government was informed about this study. A notification as an animal experiment was not necessary, as the handling of the animals did not differ from the routine handling of the fish farmers. The handling of the fish was carried out with special care and without prolonged exposure to the outside air.

### 2.2 In vivo measurement

**Figure 1:**
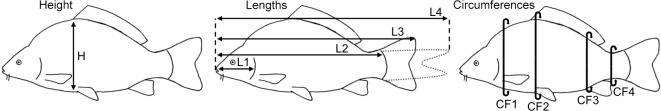
Linear measurement of carp including height, four lengths and four circumferences.

The non-sedated fish were put into narrow water-filled containers, and oxygen was added to the water. Four fish fit into one container at the same time, separated by thin walls. The carps were examined for their back-fat thickness using a mobile ultrasound device (MicroMaxx, Fujifilm SonoSite, Frankfurt am Main, Germany) and a 5 MHz endolinear probe. Water was used as the transmission medium, and therefore there was no need for direct contact between the probe and the animals. The probe was positioned underwater above the transition area from the head to back of the fish. The back-fat thickness was measured at four defined locations from two sagittal images per fish: at a distance of 2.5 cm (US1), 3 cm (US2) and 3.5 cm (US3) from the foremost point of the fat layer following its axis towards the tail fin and 2 cm (US4) towards the cranial direction from the beginning of the first ray of the dorsal fin. After the US examination, the body weight was determined and linear measurements were carried out. Linear measurements included the height, four traits of length and four traits of circumference (Fig. 1). The height (H) was measured at the maximum height of the fish, close to the level of the ventral fin. Length 1 (L1) was measured from the mouth to the gill arch, Length 2 (L2) from the mouth to the beginning of the tail fin, Length 3 (L3) from the mouth to the end of the spread tail fin and Length 4 (L4) from the mouth to the tip of the closed tail fin. Circumference 1 (CF1) was measured on the level of the pectoral fin, Circumference 2 (CF2) at the same position as the height, Circumference 3 (CF3) on the level of the anal fin and Circumference 4 (CF4) around the slimmest part of the tail fin. The fish were then slaughtered and cooled before being transported to the CT.

### 2.3 CT scanning

After slaughtering, the fish were chilled immediately using ice-cube-filled boxes. Within 6 h, whole-body scans were performed with all fish using a Siemens Somatom Plus 4 (Siemens, Germany). During one CT scan, four fish were positioned in parallel in an upright position using the above-described containers without water filling. Transversal sectional images were generated. The time required for full-body scans was 10–15 min per group of four fish depending on the length. The following settings were selected: voxel width 0.585938 mm, voxel height 0.585938 mm, slice thickness 3 mm, voltage 140 kV, rotation time 1 s, dosage 146 mA.

### 2.4 Analysis of the CT images

**Figure 2:**
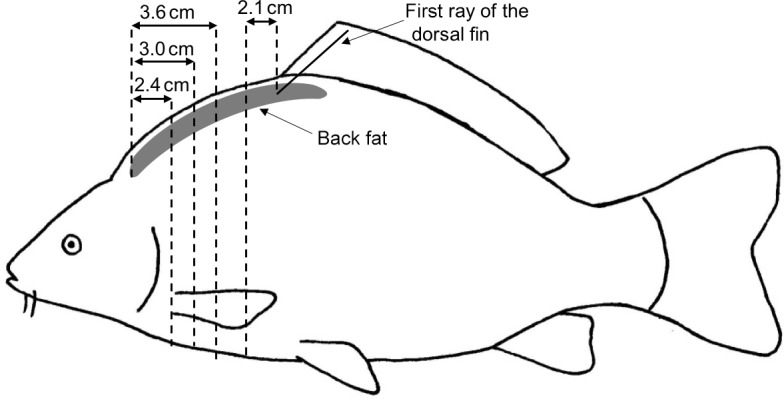
Measurement of the back-fat thickness in four positions using transversal CT images; 2.4, 3.0 and 3.6 cm from the beginning of the back fat in the direction of the tail and 2.1 cm in the direction of the head from the first ray of the dorsal fin.

**Figure 3:**
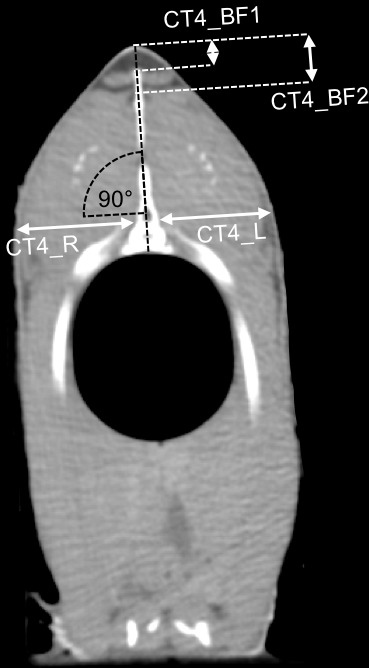
CT cross section 2.1 cm before the first ray of the dorsal fin. The back-fat thickness was measured in two different thicknesses (CT4_BF1, CT4_BF2). The thickness of the fillet was measured on the right and left side (CT4_R, CT4_L).

The CT images had a matrix of 512×512 voxels. The CT images were analyzed using the Able 3D-Doctor analysis software (Lexington, MA, USA; FDA approved). The back-fat thickness was measured using individual CT sectional images analogous to the ultrasound measuring points. The sectional plane of the US images was sagittal, as opposed to the transversal sectional plane of the CT images. The CT back-fat thickness was measured at similar positions compared to the US back-fat thickness measurement, with a deviation of ±1 mm. The CT measuring points were at a distance of 2.4 cm (CT1_BF), 3 cm (CT2_BF) and 3.6 cm (CT3_BF) from the beginning of the back towards the caudal direction and 2.1 cm (CT4_BF) towards the cranial direction from the first ray of the dorsal fin (Fig. 2). At point CT4_BF the back-fat thickness was measured twice (CT4_BF1 and CT4_BF2), as the back fat in this region is divided into two parts by a tissue (Fig. 3). The back fat is divided vertically by the spinous process, and at the upper end of the spinous process a tissue divides the fat layer horizontally. CT4_BF1 describes the thickness from the skin to the upper end of the dividing tissue. CT4_BF2 describes the thickness starting from the skin to the ventral end of the fat layer where the lean tissue begins. At CT1-3, the two-part structure of the back fat was visible for fish with high fat content, but not for lean fish. Therefore, only the smaller back-fat thickness was measured at CT1-3, analogous to CT4_BF1. In addition, the volume of the back fat was determined from the beginning of the back to the beginning of the dorsal fin. The method used for volume calculation was semiautomatic. The back-fat portion was defined as a region of interest and object boundaries including fat tissue were used to create volume rendering. To determine the fat boundaries, the tool “interactive segmentation” was used. For each fish, a specific image threshold was set based on the visual boundaries between fat and surrounding tissue. The volume of the back fat was then calculated automatically. Volume information was given in cubic centimeters.

In a next step, the fillet thickness was measured on single cross-sectional CT images at the level of CT4 in order to predict the fillet yield. At an angle of 90${}^{\circ }$ to the spinous process of the spine, the thickness of the fillet was measured at the level of the spinal canal, starting from the bone and including the skin (Fig. 3). This was done for the left and the right side of the fish (CT4_R, CT4_L).

### 2.5 Chemical analysis

After the CT scans were completed, the carps were filleted. The in vivo examinations, the slaughtering, the CT examinations and the filleting of the fish took place in 1 d. The fillets including the skin were weighed and fillet yield was calculated. The sex was determined by adspection at the opened carcass. Afterwards, the fillets were frozen at -20 ${}^{\circ }C$. For the chemical analysis, the left fillets including the skin were analyzed using Soxhlet extraction (VDLUFA, 2012). The method was modified by the investigating laboratory (Bavarian State Research Center for Agriculture – Department for Quality Assurance and Analytics, 2016). The thawed fillet including skin was mushed into a homogeneous paste; 5 g of the homogenized fillet was taken and digested with hydrochloric acid under heat and the digestion liquids were filtered. The filter residues were dried and extracted with petroleum benzine under reflux. The solvent was distilled off. The residues were dried, cooled in the desiccator and weighed. After the empty weight of the flask had been deducted, the fillet fat content was obtained. For each fillet a double analysis was carried out. The deviation between the two measurement results for the chemically analyzed fat content was less than 0.2 %. The mean value was calculated.

### 2.6 Statistics

Data were collected using Excel (version 16.15, Microsoft Corporation, Redmond, USA) and analyzed using RStudio (Integrated Development for R – RStudio, Inc., Boston, MA, USA) and MATLAB (MATLAB and Statistics Toolbox Release 2012b, The MathWorks, Inc., Natick, MA, USA). A Kruskal–Wallis test was performed to evaluate the difference in fat content between the ponds. The Mann–Whitney U test and the t test were used to evaluate the influence of sex on fillet fat content, fillet weight and fillet yield. Single linear regression models were used for the CT measurements, the ultrasound measurement, the linear measurement and the chemical analysis; multiple regression models were developed adding different linear measurements. A Bland–Altman analysis was performed in order to compare the ultrasound measurement and CT-based measurement of the back-fat thickness. The residuals were tested using diagnostic plots: residual vs. fitted plot (testing linear relationship assumptions), normal Q–Q plot (testing normal distribution), scale-location plot (testing homogeneity of variance), residuals vs. leverage plot (identify influential cases).

## 3 Results

### 3.1 Descriptive statistics

Data for body weight, fillet fat content, fillet weight and fillet yield are summarized in Table 1. The fillet weight represents the sum of the left and right fillet weight. The fillet yield was calculated as a percentage of the fillet weight of the total body weight. The fillet fat content was determined by chemical analysis of the left fillet. The fillet fat content showed a very high range, which can be attributed to the individual ponds. The average values of the fillet fat content for the carps from individual ponds were 5.2 % (±2.5, n=6), 6.0 % (±2.9, n=6), 9.3 % (±1.9, n=8), 9.6 % (±3.2, n=6), 18.7 % (±4.7, n=3) and 23.6 % (±3.4, n=4).

Both sexes were approximately equally represented (15 females, 17 males). The sex of one fish could not be determined. No significant differences between the sexes regarding the fillet fat content, the fillet weight and the fillet yield were found.

**Table 1:** Mean, minimum, maximum and standard deviation (±) for the traits body weight (g), fillet fat content${}^{*}$ (%), fillet weight (g) and fillet yield (%); n=33.

Trait	Mean	Min	Max	±
Total body weight (g)	1647	953	2607	535
Fillet fat content (%)	10.96	2.41	26.6	7.11
Fillet weight (g)	715	390	1262	265
Fillet yield (%)	42.89	38.39	48.41	2.43

${}^{*}$ Fat content of the left fillet determined by chemical analysis.

An uncertainty test of all following linear models was performed by testing the residuals using the diagnostic plots described in Sect. 2.6. The residuals showed no nonlinear patterns and were normally distributed. No major deviation was found regarding the assumption of equal variance of the residuals. One influential case was found regarding the multiple linear regression models for fillet fat content, CT-based back-fat thickness CT4_BF1 and linear measurements. However, the regression results without the influential case were only marginally better. Therefore, the fish causing the influential case was not removed in favor of the number of observations.

### 3.2 Evaluation of the fillet fat content

**Figure 4:**
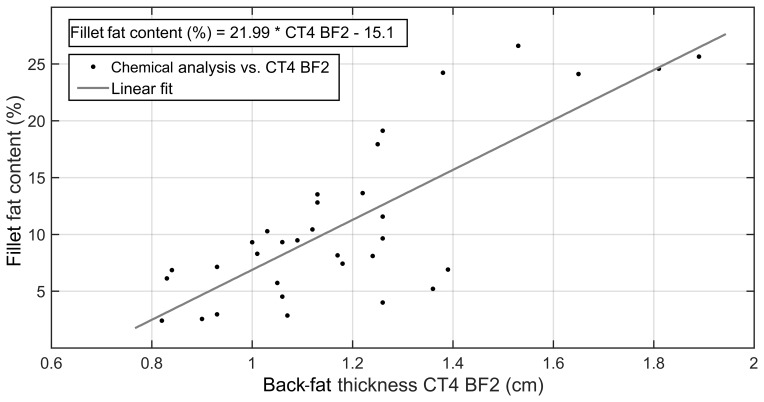
Correlation between fillet fat content determined by chemical analysis (%) and back-fat thickness CT4_BF2 (cm) determined by CT image ($R^{2}=0.62$, n=33, p<0.001).

The back-fat layer and its influence on the carcass quality, i.e., the fillet fat content, were investigated using CT technology. In a first step, the relationship between the back-fat thickness measured on CT images and the fillet fat content determined by chemical analysis was analyzed. The results are presented in Table 2. The linear correlation of CT4_BF2 and the fillet fat content determined by chemical analysis is shown in Fig. 4.

**Table 2:** Mean and standard deviation (±) of the back-fat thickness over four CT scan positions as well as the coefficients of determination ($R^{2}$) for back-fat thickness measured on a single CT image and fillet fat content measured by chemical analysis (n=33, p<0.001).

Measuring position	Mean (cm)	±	$R^{2}$	RMSE (%)
CT1_BF	0.76	0.25	0.53	4.88
CT2_BF	0.77	0.23	0.55	4.75
CT3_BF	0.77	0.22	0.55	4.75
CT4_BF1	0.63	0.15	0.31	5.92
CT4_BF2	1.19	0.25	0.62	4.41

RMSE: root mean square error, dependent variable: fillet fat content.

**Figure 5:**
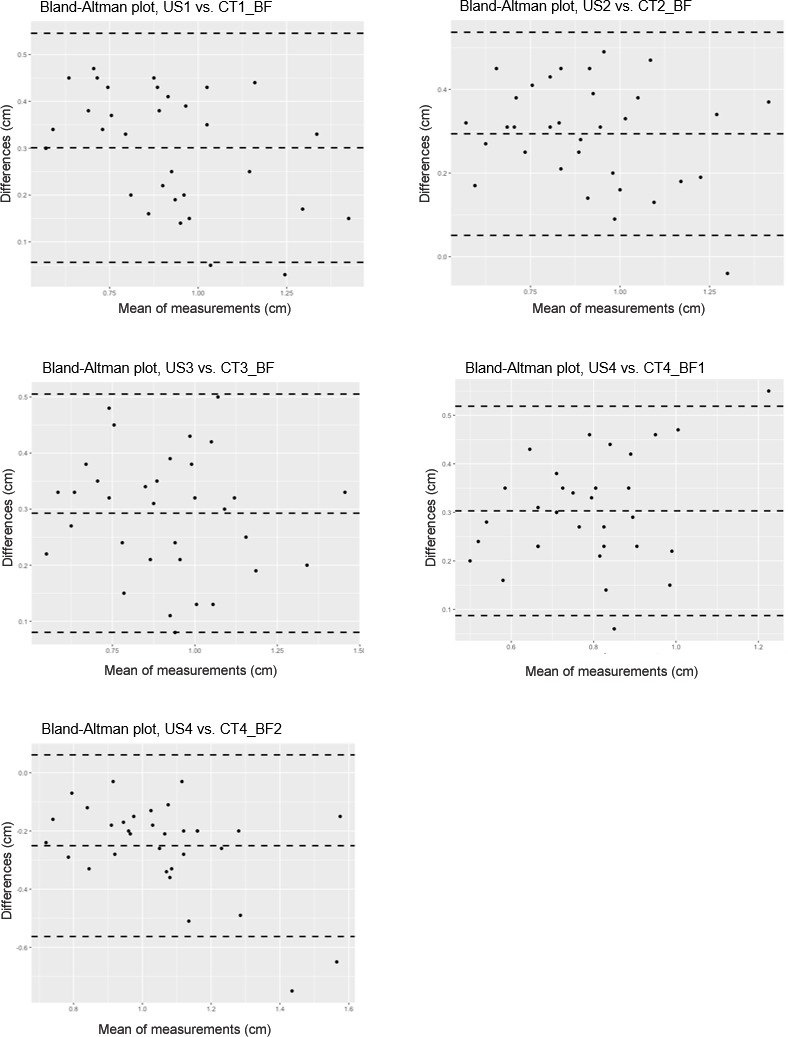
Bland–Altman analysis comparing two methods for measuring back-fat thickness in carp: the ultrasound method (US) and the CT-based method (CT_BF); n=33. Differences are calculated as US-CT, middle dashed line: mean value of the difference, upper and lower dashed lines: standard deviation of the difference.

Single linear correlations of the analogous measuring positions of CT and US are shown in Table 3. In addition, a Bland–Altman analysis was performed to compare these two measurement methods (Fig. 5).

**Table 3:** Relationship between back-fat thickness measured on a single CT image and back-fat thickness measured by ultrasound (US) (n=33, p<0.001).

CT measuring	US measuring	$R^{2}$	RMSE
position	position		(cm)
CT1_BF	US1	0.76	0.12
CT2_BF	US2	0.72	0.12
CT3_BF	US3	0.77	0.11
CT4_BF1	US4	0.64	0.09
CT4_BF2	US4	0.63	0.16

$R^{2}$: coefficient of determination, RMSE: root mean square error, dependent variable: CT-based back-fat thickness.

Next, the influence of the linear measurement was investigated. Single correlations of the results of fillet fat content determined by chemical analysis and the linear measurements are shown in Table 4.

**Table 4:** Relationship between fillet fat content (%) measured by chemical analysis and linear measurements; n=33.

Dependent variable	Independent variable	$R^{2}$	Adjusted $R^{2}$	r	p	RMSE (%)
Fillet fat content (%)	Total body weight	0.029	-0.0028	-0.169	0.348	7.01
	Length 1	0.18	0.154	-0.424	0.014	6.44
	Length 2	0.091	0.061	-0.301	0.088	6.78
	Length 3	0.093	0.064	-0.305	0.084	6.77
	Length 4	0.116	0.087	-0.34	0.053	6.69
	Height	0.031	0.000095	-0.177	0.324	7
	Circumference 1	0.00024	-0.032	-0.015	0.932	7.11
	Circumference 2	0.028	-0.0038	-0.166	0.356	7.01
	Circumference 3	0.028	-0.0038	-0.166	0.356	7.01
	Circumference 4	0.015	-0.016	-0.124	0.491	7.06

$R^{2}$: coefficient of determination, r: Pearson coefficient of correlation, RMSE: root mean square error.

In a next step, multiple regressions were performed using the results of the chemical analysis as dependent variables. As independent variables, the CT back-fat thicknesses were used and different linear measurements were added. The prediction of the fillet fat content was significantly improved with the addition of linear measurements. If more than one linear measurement was added, the prediction could not be improved decisively. The results of multiple regression analysis are shown in Table 5.

**Table 5:** Results of multiple linear regression for fillet fat content (%) and CT-based back-fat thickness (cm) with the addition of linear measurement (n=33, p<0.001).

Dependent variable	Independent variables	$R^{2}$	Adjusted $R^{2}$	RMSE (%)
Fillet fat content (%)	CT4_BF2			
	+ total body weight	0.857	0.848	2.69
	CT4_BF2			
	+ Length 1	0.789	0.775	3.27
	CT4_BF2			
	+ Length 2	0.826	0.815	2.96
	CT4_BF2			
	+ Length 3	0.818	0.806	3.04
	CT4_BF2			
	+ Length 4	0.82	0.808	3.01
	CT4_BF2			
	+ height	0.874	0.866	2.52
	CT4_BF2			
	+ Circumference 1	0.8	0.787	3.18
	CT4_BF2			
	+ Circumference 2	0.862	0.853	2.64
	CT4_BF2			
	+ Circumference 3	0.806	0.793	3.13
	CT4_BF2			
	+ Circumference 4	0.844	0.833	2.81
	CT4_BF2			
	+ all linear measures	0.894	0.839	2.31

$R^{2}$: coefficient of determination, RMSE: root mean square error.

**Figure 6:**
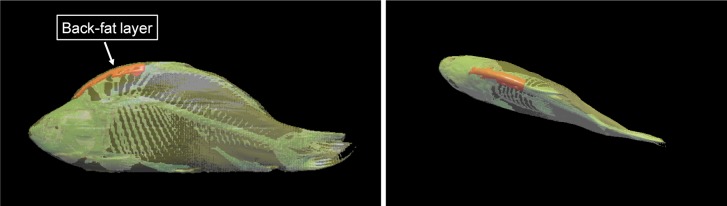
3-D model of a mirror carp created with 3D-Doctor software showing the back fat from the beginning of the back to the beginning of the dorsal fin.

Furthermore, the volume of the back fat (${\mathrm{cm}}^{3}$) was calculated, starting from the beginning of the back until the beginning of the dorsal fin. Figure 6 shows a 3-D model of one of the examined carp representing the back-fat layer. The mean volume (n=33 fish) of the back-fat layer was 7.62 ${\mathrm{cm}}^{3}$, with a standard deviation of ±5.40; the minimum was 1.03 ${\mathrm{cm}}^{3}$ and maximum 20.64 ${\mathrm{cm}}^{3}$. Single correlation with the chemical analysis generated an $R^{2}$ of 0.85. In a next step, multiple regressions with the linear measurements were carried out (Table 6).

**Table 6:** Results of multiple linear regression for fillet fat content (%) and back-fat volume (${\mathrm{cm}}^{3}$) with the addition of linear measurement (n=33, p<0.001).

Dependent variable	Independent variables	$R^{2}$	Adjusted $R^{2}$	r	RMSE (%)
Fillet fat content (%)	Back-fat volume	0.85	0.846	0.9222	2.75
	Back-fat volume				
	+ total body weight	0.899	0.892	0.9481	2.26
	Back-fat volume				
	+ Length 1	0.888	0.88	0.9422	2.38
	Back-fat volume				
	+ Length 2	0.894	0.887	0.9454	2.32
	Back-fat volume				
	+ Length 3	0.894	0.887	0.9454	2.32
	Back-fat volume				
	+ Length 4	0.892	0.885	0.9445	2.34
	Back-fat volume				
	+ height	0.893	0.885	0.9447	2.33
	Back-fat volume				
	+ Circumference 1	0.905	0.899	0.9515	2.19
	Back-fat volume				
	+ Circumference 2	0.892	0.885	0.9444	2.34
	Back-fat volume				
	+ Circumference 3	0.889	0.881	0.9427	2.37
	Back-fat volume				
	+ Circumference 4	0.896	0.889	0.9465	2.3
	Back-fat volume				
	+ all linear measures	0.923	0.883	0.9609	1.97

$R^{2}$: coefficient of determination, r: Pearson coefficient of correlation, RMSE: root mean square error.

### 3.3 Evaluation of the fillet yield

The fillet yield was calculated from the fillet weight and the total body weight of the fish. Mean fillet yield was 42.89 % (±2.43), with a maximum fillet yield of 48.41 % and a minimum fillet yield of 38.39 %.

The fillet thickness measurement was performed on single CT images (Fig. 3). The mean of CT4_R was 2.74 cm (±0.32), and the mean of CT4_L was 2.77 cm (±0.33). The two measurements CT4_R and CT4_L were added up (CT4_R + CT_4_L).

In order to predict fillet yield, linear regression studies were done. In a first step, the total body weight and the linear measurements such as lengths, height and circumferences were used to predict the fillet yield. In a next step, linear regression studies were done for fillet yield and the CT measurement (Table 7). Multiple regression models of the CT measurement combined with linear measurements did not result in stronger predictions.

**Table 7:** Results of single linear regression between fillet yield (%), linear measurement and CT measurement; n=33.

Dependent variable	Independent variable	$R^{2}$	Adjusted $R^{2}$	p	RMSE (%)
Fillet yield (%)	Total body weight	0.42	0.41	<0.001	1.84
	Length 1	0.11	0.08	0.0578	2.29
	Length 2	0.34	0.32	<0.001	1.98
	Length 3	0.35	0.33	<0.001	1.96
	Length 4	0.3	0.28	<0.001	2.03
	Height	0.37	0.34	<0.001	1.94
	Circumference 1	0.47	0.45	<0.001	1.78
	Circumference 2	0.38	0.36	<0.001	1.91
	Circumference 3	0.28	0.25	0.0017	2.07
	Circumference 4	0.42	0.4	<0.001	1.85
	CT4_R	0.42	0.4	<0.001	1.85
	CT4_L	0.46	0.44	<0.001	1.79
	CT4_R + CT4_L	0.46	0.42	<0.001	1.8

$R^{2}$: coefficient of determination, RMSE: root mean square error.

## 4 Discussion

The study aimed at the prediction of the body composition of mirror carps using CT and linear measurements. A sample size of n=33 fish was examined. During this study, no effects of sex on carcass composition were observed. On the one hand, Hancz et al. (2003a), Fajmonova et al. (2003) and Varga et al. (2013) found no influence of sex on the body composition of carp (sample size: n=18, n=48 and n=80). Kocour et al. (2007), on the other hand, investigated a larger number of carp (n=331) and found a significant influence of sex, resulting in females being larger and fatter than males.

However, carcass composition is not only determined by environmental factors; it also depends on the breed (Gela et al., 2003; Varga et al., 2013). Genetic improvement in common carp was investigated by several authors (Bakos and Gorda, 1995; Linhart et al., 2002; Kocour et al., 2005, 2007). The best results for carcass quality were achieved by cross-breeding. However, before genetic selection can be performed, a precise phenotyping must be carried out.

### 4.1 Evaluation of the fillet fat content

The fillet fat content of the examined carps ranged between 2.41 % and 26.60 %, measured by chemical analysis. In addition, the fat content of the carps varied considerably between the different ponds the fish originated from, which is mainly related to feeding, but also to other factors, e.g., stocking density and water temperature (Zeitler et al., 1984; Yamamoto et al., 2003; Schwarz et al., 2006).

The back-fat layer is known to be a good predictor of the fat content of carp (Oberle et al., 2015). It is of constant thickness in the range 2.4 to 3.6 cm behind the beginning of the back. The back fat enlarges in front of the dorsal fin in our examined fish. Hancz et al. (2003a) described the area around the beginning of the dorsal fin as the richest in fat and calculated the fat area (${\mathrm{cm}}^{2}$) in their study using CT.

In the area of the dorsal fin the back fat is split. The spinous process of the spinal column protrudes from below into the fat layer. At its upper end a muscular tissue divides the fat layer into two sections. This separation made it difficult to measure the thickness of the back-fat layer accurately in the area of the dorsal fin using in vivo methods such as ultrasound.

The CT-based back-fat thickness measurement and the US measurement showed higher correlation in the range of 2.4 to 3.6 cm towards the caudal direction (r=0.85–0.88 with an RMSE of 0.11–0.12 cm) than in the region of the dorsal fin (r=0.79–0.80 with an RMSE of 0.09–0.16 cm). The Bland–Altman analysis showed mainly positive differences, which leads to the conclusion that the US-based method provides higher measurement results than the CT-based method.

Using single linear regression models to evaluate the correlation between fillet fat content and linear measurement, moderate, nonsignificant results were obtained. The RMSE ranged between 6.44 % and 7.11 %. Remarkably, the best result was obtained by predicting the fat content using Length 1; the Pearson correlation coefficient was negative (r=-0.42). In a previous study examining a larger sample of mirror carps, significant inverse correlations between fillet fat content and linear measurements were found (r of -0.50 to -0.57, n=250) (Maas et al., 2019).

The back-fat thickness measurement using CT images showed a correlation of r=0.55 to 0.78 with regard to the fillet fat content. The RMSE ranged between 4.41 % (CT4_BF2) and 5.92 % (CT4_BF1). The Pearson coefficient of correlation could be improved by adding linear measurement. Approximately 93 % of the variation in fillet fat content could be explained by the back-fat thickness combined with one linear measurement (height of the fish, circumference on the level of the height or total body weight).

The volume calculation of the back fat provides better results with regard to the fillet fat content than the thickness measurement. The evaluated area ranged from the beginning of the back to the beginning of the dorsal fin. The prediction of the fillet fat content using the back-fat volume resulted in a Pearson coefficient of correlation of 0.92 (RMSE of 2.75 %). A Pearson coefficient of correlation of 0.94–0.96 was achieved using the chemical analysis results as a dependent variable and including back-fat volume and linear measurement.

Hancz et al. (2003a) achieved a similar result in calculating the back-fat area (${\mathrm{cm}}^{2}$) in common carp ($R^{2}=0.88$). Romvári et al. (2002) calculated the fat volume in common carp, grass carp, silver carp and pike perch using CT images from the end of the operculum to the beginning of the caudal fin. The fat content of pike perch could not be evaluated because it was extremely low. For the cyprinid species, an $R^{2}$ of 0.93 was generated using CT-based fat volume and the chemical fat content of the fillet. The method used by Hancz et al. (2003a) and Romvári et al. (2002) was based on a CT study (Romvári et al., 1998) on rabbits. The HU density range from -90 to +160 was calculated for fat and lean tissue. A total of 10 HU values were combined, resulting in 25 HU variables used to calculate a “fat index” and to generate prediction equations for the fat content. Kolstad et al. (2004) provided an r of 0.95 in Atlantic halibut by calculating the relationship between fat deposit areas (in %) measured in one sectional CT image and chemical fat content measured in cutlets. For fat, the HU range was set between -174 and -18 HU. The mentioned studies calculated the fat area or fat volume using the Hounsfield unit (HU) range. One criticism of this method, however, is the incomplete separation of fat and lean tissue, since some voxels are not clearly assignable but consist of a mix of tissues (Luiting et al., 1995). In our study, the volume was calculated semiautomatically by manual limitation of the dorsal fat layer. The accuracy of our method of volume calculation compared to the HU method should be investigated in a next step.

### 4.2 Evaluation of the fillet yield

Usually, carp is marketed as whole fish. The marketing of fillets as part of a new food trend is becoming increasingly important. Therefore, besides the fillet fat content, the fillet yield (%) is an important parameter in the marketing of carp. Compared to other studies, the fillet yield of 42.89 % found in our sample was very good. Fillet yields including the skin were examined in Austria with 34 %–35.9 % (Bauer and Schlott, 2009), in France with 34.6 % (Cibert et al., 1999) and in the Czech Republic with 41.1 % (Kocour et al., 2007).

Linear regressions between fillet yield and linear measurements resulted in a Pearson coefficient of correlation of 0.33–0.68 and RSME of 1.78 %–2.29 %. The best correlation was achieved between fillet yield and the circumference of the fish on the level of the pectoral fin (CF1). With the exception of Length 1 and Circumference 3, all linear measurements achieved a significant correlation with the fillet yield (p<0.001). For comparison, Bauer and Schlott (2009) found neither a correlation between fillet yield and body weight nor between fillet yield and total length in Austrian carp (n=90). Kocour et al. (2007), however, found a correlation of 0.43 between fillet yield and body weight and a correlation of 0.46 for fillet yield and standard length in carp (n=331). Kocour et al. (2007) also investigated the breed of the examined carp.

Linear regressions between fillet yield and CT measurements of fillet thickness resulted in a Pearson coefficient of correlation of around 0.67 with RMSE of 1.79 % to 1.85 %. Kolstad et al. (2004) found correlations of 0.53 to 0.95 by calculating the area (%) of lean tissue in different CT scan positions in Atlantic halibut (n=50). The fish were gutted before the CT was done. In our study, the carps were not gutted before they were scanned by CT. Therefore, a clear separation of fillet and innards could not be determined on the CT images, and an area or volume calculation of the fillet was not possible.

## 5 Conclusion

In summary, it can be concluded that a volume calculation of the back fat based on three-dimensional CT images provides a more accurate prediction with regard to the fillet fat content than two-dimensional measurements of the back fat using single CT images or ultrasound.

The back fat in carp has proven to be a significant area regarding the fillet fat content and therefore the carcass quality. Multiple linear regression models including linear measurements can be used in both 2-D and 3-D measurements to improve the Pearson coefficient of correlation.

The fillet yield can be predicted with moderate results by measuring the thickness of the fillet on single transversal CT images. A prediction on a similar level is provided by some selected linear measurements and by total body weight.

In principle, CT technology, combined with linear measurements, offers great potential for phenotyping carp. In addition, the CT images can be used in the long term to evaluate further parameters for predicting body composition in mirror carp.

The results of our study should be verified with a larger number of animals. Next, the best predictive model could be established for in vivo measurements and will help to select suitable fish for breeding. In this way, a system of quality-oriented production can be established that leads to a high-quality product and thus to a high level of consumer acceptance.

## Data Availability

The original data are available upon request to the corresponding authors.
